# Retrospect of the Medical Sciences

**Published:** 1843-06-10

**Authors:** 


### RETROSPECT OF THE MEDICAL SCIENCES.

### ON THE SPECIAL FUNCTION OF THE SKIN.

BY R. WILLIS, M. D.

The purpose which is answered in the animal economy by the function of the skin has not been precisely indicated by physiologists. It is universally acknowledged of immense importance to the health; but no one has said how or wherefore it is so. The chemical analysis of the sweat is acknowledged to throw no light on the ends for which it exists; and the principal matter excreted from the skin is water: sweat contains about 99 per cent. of this fluid. The author of this paper considers the water as the essential excretion; not, however, in the usual sense in which the word excretion is used, nor yet as a means of regulating or reducing temperature. In the means which nature takes to preserve the lower animals from the effects of loss of heat by sending them into the world ready clothed, and in the uniformity with which man contrives to clothe himself over so large a portion of the earth's surface, he sees that the object is generally to economise heat, not to dissipate it. He says there is even something like an absurdity involved in the invocation of a system to cool the body, seeing that one great business of life, in all the temperate and cold countries of the world, is to guard against the loss of heat. Heat is lost, indeed, to a great extent, in converting the product of the sudoriparous glands into vapour; but the loss here was unavoidable; it is not the end. In spite of all the sudoriparous glands can do, also, the experiments of Delaroche and Berger have shown that the heat of the body rises considerably above the standard when exposed to temperatures somewhat higher than itself. In chambers heated to 120° and 130° Fahr., the temperature of animals rises, within an hour, to from 11° to 16° above what it had been before, and life is destroyed. And then the suppression of the cutaneous exhalation is by no means followed by a rise in the temperature of the body. In general dropsies, which are attended with a remarkable diminution of this secretion, an icy coldness usually pervades both the body and the limbs. A great fall in the animal temperature was likewise found by Fourcault and Becquerel and Breschet to to be the effect of covering the body with a varnish impervious to perspiration; and so serious was the general disturbance of the functions in these circumstances, that death sometimes ensued in the course of from three to four hours. Delaroche and Berger had also found that confinement in an atmosphere of no higher temperature than the body of an animal, but saturated with moisture, was rapidly fatal; animals scarcely resisted such an atmosphere for an hour before they were reduced to extremity. Whatever prevents the skin from performing its function, *i. e.* from throwing off a little water, is very speedily fatal.

How may this effect be produced? How does it happen that health and even life can be so immediately dependent, as they certainly are, on the elimination of some thirty-three ounces from the general surface of the body in the course of the twenty-four hours? To this the author answers,—by securing the conditions which are necessary for the endosmotic transference between arteries and veins of the fluids that minister to nutrition and vital endowment.

It is admitted by physiologists that the blood, while still contained within its conducting channels, is inert with reference to the body, no particle of which it can either nourish or vivify until that portion of it which has been denominated the *plasma* has transuded from the vessels, and come into immediate contact with the particle that is to be nourished and vivified: but no physiologist has yet pointed out the efficient cause of these tendencies of the plasma, first, to transude through the wall of its efferent vessels, and secondly, to find its way back again into the afferent conduits. The explanation given by the author is, that in consequence of the out-going current of blood circulating over the entire superfices of the body, perpetually losing a quantity of water by the action of the sudoriparous glands, the blood in the returning channels has thereby become more dense and inspissated, and is brought into the condition for absorbing, by endosmosis, the fluid perpetually exuding from the arteries, which are constantly kept on the stretch by the injecting force of the heart.

Repeated experiment has demonstrated the fact that the blood in the veins is of somewhat greater density than that in the arteries. If the specific gravity of arterial blood be allowed to be 1,050, that of venous blood in the mean will be 1,503.

In an appendix to the paper, the author points out a few of the practical applications of which the above-mentioned theory is susceptible. Interference with the function of the skin, and principally through the agency of cold, he observes, is the admitted cause of the greater number of acute diseases to which mankind, in the temperate regions of the globe, are subject. He who is said to have suffered a chill, has, in fact, suffered a derangement or suppression of the secreting action of his skin, a process which is altogether indispensable to the continuance of life; and a disturbance of the general health follows as a necessary consequence. Animals exposed to the continued action of a hot, dry atmosphere die from exhaustion; but when subjected to the effects of a moist atmosphere of a temperature not higher than their own, they perish by the same cause as those which die from covering the body with an impervious glaze; for, in both cases, the condition required for the access of oxidized, and the removal of deoxidized plasma, are wanting, and life necessarily ceases. The atmosphere of unhealthy tropical climates differs but little from the vapour-bath at a temperature of between 80° and 90° Fahr.; and the dew point in those countries, as, for example, on the western coast of Africa, never ranges lower than three or four degrees, nay, is sometimes only a single degree, below the temperature of the air. Placed in an atmosphere so nearly saturated with water, and of such a temperature, man is on the verge of conditions that are incompatible with his existence: conditions which may easily be induced by exposure to fatigue, to the burning sun, or other causes which excite the skin while the circumstances necessary to the exercise of its natural function are wanting. The terms *Miasma* and *Malaria* may, according to the author, be regarded as almost synonymous with air at the temperature of from 75° to 85° Fahr., and nearly saturated with moisture.

The secretion of the skin otherwise suppressed is attended with no less fatal consequences. What kills a patient within the first 48 hours of a bad attack of scarlet fever? The autopsy shows nothing amiss. He dies undoubtedly from the complete suppression of the function of the skin. Restore this, and he will do well; all the stimulants in the world will not rouse him if the function of his skin be not restored. —*Proceedings of the Royal Society*, March 2d and 9th, *from London Medical Gazette.*

PHYSIOLOGY AND PATHOLOGY OF THE BLOOD.

[The following is taken from an excellent paper by Dr. Rees, contained in the last number of "Guy's Hospital Reports."]

The coloring matter of the blood and its true relation to the corpuscle is a point which has hitherto been but little understood, and on this subject Dr. Rees first treats. He observes, "Much confusion has arisen from the terms red particle, red globule, and red corpuscle being regarded as synonymous with hæmatosine or coloring matter, the latter expression meaning, in its correct signification, nothing more than one of the constituents of the red corpuscles; there being a white matter also present in those bodies, which chemists have only noticed within the last few years, and have never yet ventured to define more particularly than as the white matter of the corpuscles." By a series of microscopic experiments, Dr. R. has demonstrated that the red corpuscles are closed sacs containing a fluid within them, which fluid is itself of a red color, the investing sac being white—a fact which he arrived at by bursting the blood corpuscles by the addition of water, and then separating the contents of the corpuscles from the containing membrane. The membranes of the blood corpuscles, notwithstanding their extreme tenuity, possess the property of admitting the passage of fluids by endosmosis—a fact which is established by submitting the corpuscles to the action of fluids of higher and lower specific gravity, by which the flaccidity or distension of the globules was produced.

With regard to the condition of the iron, and its true position in the organization of the corpuscle, Dr. R. has shown, by an extension of the experiment before alluded to, that the iron is contained in the red coloring fluid, and not in any other of the constituent parts of the corpuscle. From the clear solution obtained after the bursting of the corpuscle by the addition of distilled water, a dry mass was by evaporation procured, by incinerating which it was found to contain the whole of the iron of the corpuscles, whilst no iron is contained in the sacs or cases of the latter, or in their nuclei. In alluding to the nature of the smaller corpuscles, which are constantly found floating with those of larger size, and which by many microscopists have been supposed to be immature corpuscles, the author observes, "It must be a necessary part of the process by which these small corpuscles arrive at maturity, that iron enters within the envelope, to supply one of the constituents of the red coloring matter; and to ensure this effect two conditions are required. Firstly, a liquor containing iron in solution must be applied to the membrane of the corpuscle; and, secondly, that this liquor must be of a specific gravity less than that contained in the corpuscle, or it will not enter it in quantity. Both these required conditions are to be found as physico-chemical characteristics of the mixture of chyle and lymph which enters the blood by the thoracic duct, to which fluid all experimenters have given a lower specific gravity than the liquor sanguinis. Thus the specific gravity of the contents of the thoracic duct in the human subject, which I lately analysed, was 1024, while that of the liquor sanguinis may be given at 1052 at the least. From other experiments on the cat, dog, and the ass, I am satisfied of the general truth of this statement. The iron which exists in the chyle is not contained in the crassamentum which forms by coagulation; but we find it, on the contrary, as a constituent of the serum in a perfect state of solution, so that it may enter with facility through the membranes of the corpuscles; so perfect, indeed, is this solution, that, even after evaporating the chyle to dryness, we are enabled to extract the iron from the albuminous matters by digestion in water. It exists, in fact, dissolved in that constituent of the chyle which is called the aqueous extractive, and most probably in the form of lactate. Nature has then in this admirable manner provided for the introduction of iron into the corpuscle, by presenting it in a perfectly soluble form to the enveloping membrane, and dissolved in a liquor of a specific gravity suited to effect the necessary endosmodic actions."

The author next proceeds to point out certain pathological phenomena which may be expected to result when the due balance and arrangement of the physical properties of the fluids become destroyed. He regards the healthy condition of the membrane of the blood corpuscles, by which transmission of liquors and gases may take place, as being of the same importance to the maintenance of life as is the previous condition of the respiratory tubes, and that life would be equally destroyed by the loss of those special properties in the former as by the closure of the latter. "Let the blood become destroyed, so that its specific gravity is lessened, and we may feel assured that if the physical qualities, more especially the specific gravity of the chyle, be not simultaneously affected, and that, too, in a due proportion, the result must be that the red coloring matter, the great oxygenator of the blood, is no longer produced in its ordinary quantity—the ferruginous serum of the chyle not being able to enter the blood corpuscle as in health. If the degeneration above alluded to take place, we must recollect that all the solids of the body through which the blood courses are formed with pores and of materials admitting of endosmodic action; and that it is impossible for the solid constituents to preserve their health if constantly acted upon by the blood at a specific gravity of 1030 to 1036, instead of 1052 to 1057—the equilibrium of health being no longer preserved, and the watery blood inducing a like condition in the other solids."

The author expresses his desire to direct the attention of the profession to the study of endosmosis as applied to pathology, and his belief that many of the phenomena of Bright's disease are attributable to the condition of blood noticed in his essay.

ANÆMIA OF THE BRAIN INDUCED BY FATAL HEMMORRHAGE.

BY GEORGE BURROWS, M. D.

It may appear surprising that Dr. Kellie has been so often quoted as asserting the brain cannot be depleted by blood letting; when we find him stating, his experiments satisfactorily proved that these brains had really been depleted by bleeding, and their vessels drained of a very sensible proportion of the blood usually contained in them. But in opposition to the conclusions drawn from these experiments, we find, in a subsequent communication to the Medico-Chirurgical Society of Edinburgh, Dr. Kellie affirming "that, in the ordinary state of the parts, we cannot lessen, to any considerable extent, the quantity of blood within the cranium by arteriotomy or venesection;" whereas, if the skull of an animal be trephined, then hæmorrhage will leave very little blood in the brain.

This apparent contradiction between the results of experiments and subsequent statements induced me to repeat the experiment of bleeding animals to death, and comparing the state of the cerebral blood-vessels in them and in animals which had died from other causes.

In justice to myself I wish to state that, if I could have found any series of experiments, performed by others, which corroborated or invalidated the opinions of Drs. Abercrombie and Kellie, I should have refrained from the needless repetition of similar experiments on living animals. I have, however, up to the present time, fruitlessly searched for any additional information on this interesting point in physiology, a point which has such direct bearings on practical medicine. I had anticipated finding in Dr. Marshall Hall's work "On the Effects of Loss of Blood," the desired information; but it does not appear that, at the time of the publication of that volume, the author had made any examination into the state of the blood-vessels of the brain after hæmorrhage; for he remarks "that we are altogether in want of a series of observations on the effects of loss of blood on the internal organs." Thus disappointed in my search for information on this subject, I determined to resort to fresh experiments.

On the 11th of January, 1843, I killed two well-grown rabbits. The one (A) by opening the jugular vein and carotid artery on one side of the throat; the other (B) was strangled. Each animal died violently convulsed. A ligature was drawn tightly round the throat of the rabbit (A) immediately it expired, to prevent any further escape of blood from the vessels of the head. The rabbits were allowed to remain twenty-four hours on a table resting on their sides.

While the blood was flowing from the rabbit (A), the conjunctiva was observed to become pallid, and the eyeballs to shrink within the sockets. Upon the examination of the head of this rabbit, the integuments and muscles appeared blanched and exsanguined. Upon removing the upper portions of the cranium, the membranes of the brain were found pallid, and scarcely the trace of a blood-vessel was to be detected on the surface of the brain. The longitudinal and lateral sinuses were nearly empty of blood, and their course was not denoted by any colour of blood. Upon making sections of the brain, the interior appeared equally exsanguined.

Soon after the cord was drawn tight around the throat of the rabbit (B), the conjunctiva became congested, the eyeballs turgid, prominent, and even projecting beyond the margin of their sockets. The integuments and muscles of the head were found full of blood. Upon opening the cranium, the superficial vessels of the membranes, as well as the sinuses, were full of dark liquid blood. The whole substance of this brain, and its membranes, appeared of a dark reddish hue, as if stained by extravasated blood.

The contrast between the two brains in point of vascularity, both on the surface and the interior, was most striking. In the one scarcely the trace of a blood-vessel was to be seen; in the other every vessel was turgid with blood. It seems hardly necessary to bring forward further evidence to prove that death by hæmorrhage has a most decided effect in depleting the vessels, and reducing the quantity of blood within as well as upon the outside of the cranium.

I have, however, repeated the experiments with similar results. In fairness to Dr. Kellie I should state, that I have attended at the slaughtering of sheep by butchers, and find the brains of those animals much less depleted than the brains of rabbits which have died by hæmorrhage. But these sheep did not die from simple loss of blood; but partly from division of the pneumogastric nerves, and cervicle portion of the spinal cord. These lesions, no doubt, influenced the appearances.

Hence it is not a fallacy, as some suppose, that bleeding diminishes the actual quantity of blood in the cerebral vessels. By abstraction of blood we not only diminish the momentum of blood in the cerebral arteries, and the quantity supplied to the brain in a given time, but we actually diminish the quantity of blood in those vessels. Whether the vacated space is replaced by serum, or resiliency of the cerebral substance under diminished pressure, is another question, into which I do not now enter.

2dly.—Dr. Kellie, assuming the cranium to be a perfect sphere, proceeds to show that the quantity of blood in the cerebral vessels is not affected by posture.

"I think," writes Dr. K. "it quite certain, at least in a previously sound and healthy condition of the brain and its vessels, no change of posture can impel into, or confine more or less blood within those vessels than naturally belongs to them; though I am willing to allow that the general pressure of the circulating fluid may in this way be, under certain circumstances, increased or diminished, and the circulation through the head accelerated, retarded, or disturbed."

In order to ascertain, as far as such an experiment can do, the total effect of the gravitation of the blood upon the vessels of the brain, Dr. K., immediately after administering a destructive dose of prussic acid to two dogs, suspended the one by the heels, and the other by the ears. He allowed them to remain thus suspended for 18 hours, when they were taken down for examination.

The effects of posture on the parts exterior to the skull Dr. K. reports to be very great. In the former animal the integuments and their vessels were filled and congested to the greatest possible degree; the integuments of the head of the second dog were pale, and the vessels empty. "Within the head," continues Dr. K., "the contrast was but trifling. The sinuses beyond all doubt were loaded in the first case, and rather empty in the other; the difference of appearance in other parts of the brain was but little striking." Dr. Kellie's own words, as to the condition of the sinuses in the two animals, assured me that posture had a much greater effect on vascular congestion of the brain than he was willing to admit. I therefore repeated his experiment.

On the 28th of December, 1842, two full-grown rabbits were killed by prussic acid, and, while their hearts were still pulsating, the one (C) was suspended by the ears, the other (D) by the hind legs. They were left suspended for 24 hours; and, before they were taken down for examination, a tight ligature was placed round the throat of each rabbit, to prevent, as effectually as was possible, any further flow of blood to or from the head, after they were removed from their respective positions.

In the rabbit (C) the whole of the external parts of the head, the ears, the eye-balls, &c. were pallid and flaccid; the muscles of the scalp and bones of the cranium were also remarkably sanguined. Upon opening the cranium, the membranes and substance of the brain were pallid, the sinuses and other vessels were exsanguined; anæmic beyond my expectation.

In the rabbit (D) the external parts of the head, the ears, eye-balls, &c. were turgid, lived, and congested. The muscles and bones of the cranium were of a dark hue, and gorged with blood, which at some parts appeared extravasated. Upon opening the cranium, the membranes and vessels were dark and turgid with liquid blood; the superficial veins were prominent, the longitudinal and lateral sinuses were gorged with dark blood, and there was staining of the tissues, if not extravasation of blood into the membranes. The substance of the brain was uniformly dark, and congested to a remarkable extent.

Dr. Kellie asserts, but I think his experiments do not support him, that the contrast in the appearances within the heads of the two animals was but trifling. In my analogous experiments the contrast was most striking. In the one was to be seen a most complete state of anæmia of the internal as well as external parts of the cranium; in the other a most intense hyperæmia or congestion of the same parts; and these opposite conditions in the vascularity of the brain induced solely by posture, and the consequent gravitation of the blood.

If the cranium were the perfect sphere, as taught by Monro, and as subsequently maintained by Abercrombie and other distinguished writers on the pathology of the brain, these effects on its circulation (which I have now exhibited) ought not to have resulted from the force of gravity on the blood in the cerebral vessels.

From the foregoing experiments it would appear, that the principle of the subsidence of fluids after death operates on the parts contained within the cranium, as well as upon those situated in the thorax or abdomen.—*Lond. Med. Gaz.* April 28.

---

### STRUMOUS PERITONITIS.

The March number of the "Dublin Journal of Medical Science" contains communications from Sir Henry Marsh and Dr. Fleetwood Churchill, on a form of peritonitis occurring in persons of a strumous diathesis, to which the above title has been given. Strumous inflammation of the peritoneum with effusion may present itself either as an acute or chronic disease; the latter, which is the more frequent form, may be an evident consequence of the former, or it may occur without our being able to recognise any preceding acute stage, coming on so gradually, in fact, that the nature of the complaint may not be discovered until it is fully developed. As M. Duges observes, "there may be occasional pains, colics, irregular attacks of diarrhœa, emaciation, paleness, for weeks or even months before the disease is established, while from the earlier and more prominent symptoms being referable to the mucous membrane of the intestinal canal, the real affection may be overlooked, and the fatal results attributed to the diarrhœa.

Bad diet, cold, privations, excesses, dentition, constipation, &c. have been mentioned as exciting causes of this variety of peritonitis; but the principal one, in Dr. Churchill's opinion, is an extension of irritation from the intestinal mucous membrane. It also occurs as one of the sequelæ of febrile diseases, such as scarlatina, measles, &c. Sir H. Marsh observes, that it frequently arises without the intervention of any well marked exciting cause, and sometimes sets in either gradually or abruptly, during the progress of some other disease. The age most obnoxious to its supervention is between early childhood and puberty, and for a few years after that period; it is rarely met with in infancy or after the age of thirty. It is almost confined to children of a strumous habit and lymphatic temperament; and is frequently complicated with, and is perhaps a consequence of, mesenteric disease.

The symptoms of the acute form are, wandering pains in the abdomen, increased on pressure, and gradually becoming more severe, with hot and dry skin, scanty, high-colored urine, small and very frequent pulse, and extreme distress and anxiety, rapidly followed by effusion into the cavity of the peritoneum. In the chronic variety the mode of invasion varies widely; in one class of cases the patient labours under diarrhœa for a considerable time with or without pain; the appetite is pretty good, the temperature natural, and the pulse quiet; but at length—it may be for weeks or months—complaints are made of a sensation of pricking, or of paroxysms of pain and a feeling of tightness in the abdomen, which, upon examination, is found to be more or less swollen. In other cases there is a certain amount of pain from the beginning, occurring in paroxysms with perfect intervals, and though at first limited to one part of the abdomen, yet by degrees spreading over and occupying the whole; or again, there may not be any complaint of pain, merely a feeling of distension, with variable appetite, irregular bowels, and progressive emaciation. In many cases, indeed, the early symptoms are so slight that no attention is paid to them until the emaciated appearance of the patient excites alarm. The abdomen, on examination, is probably found tumid, and in some degree tender in parts. Dr. Gregory states that tenderness on pressure is probably felt from the commencement. As the disease advances, the patient complains of pain occurring in paroxysms, beginning in some one part of the abdomen, and gradually spreading over the entire, and increasing in frequency and in severity according to the duration of the disease. This state is accompanied by a certain amount of tenderness on pressure, and more or less uneasiness in walking or even standing erect. This is followed by a feeling of distension and enlargement of the abdomen, with fluctuation more or less evident on careful percussion. The enlargement of the abdomen is not always equable; in some cases, especially in the commencement, the umbilical region protrudes. As the effusion increases, the entire abdomen enlarges, loses its softness, and becomes tense and hard, though occasionally unequally so, and its integuments hot and dry. In some rare cases the intestinal canal preserves its integrity for a long time; the tongue is pretty clean, the appetite much as usual, the bowels regular, or perhaps rather constipated; but in the large majority of cases we find the tongue white, loaded, and flabby; more or less thirst; the appetite irregular and fastidious, sometimes increased, more frequently impaired or lost altogether; the bowels relaxed or constipated, perhaps alternately; the stools fœtid, and of whitey-brown or bluish color. The quantity passed sometimes in the twenty-four hours, without the aid of medicine, is enormous. Dyspnœa may be induced either by the quantity of the abdominal effusion or by accompanying hydrothorax; the pulse is increased in frequency, but diminished in strength and fulness; the urine is scanty, the skin hot, and there are distinct evening exacerbations of a fever of the hectic character. It is hardly necessary to add that so formidable and long-continued a disease is attended with great emaciation and exhaustion. As it progresses, the local symptoms are aggravated; the quick pulse and fever, with exacerbations, more remarkable; the weakness and incapability of exertion more extensive; the patient, in short, is utterly worn ont.

The course of the disease is generally very long; it may terminate, first in resolution, secondly, in a circumscribed collection of the effused fluid and its final evacuation with more or less subsidence of the original affection, and, thirdly, in death. The majority of the cases terminate fatally at different intervals from the commencement of the attack. Instead of diminishing, the symptoms progressively increase in intensity. The abdomen becomes very tense and tender, the fever high, the pulse very quick and feeble, the thirst considerable, the diarrhœa persistent, the exacerbations severe, the emaciation and exhaustion extreme. The countenance becomes sunken, the extremities cold, the surface covered with a clammy sweat, and occasionally dotted with petechiæ, and at length, after a prolonged period of suffering, death closes the scene. The appearances presented after death are, more or less effusion into the peritoneal cavity, with shreds of lymph floating therein. The intestines are agglutinated together, and sometimes to the peritoneum, which membrane is occasionally thickened and partially injected, and sometimes studded with miliary tubercles, or has tubercular matter deposited on it. The mesenteric glands may be healthy, or they may be enlarged, and contain tubercular matter. In two of Sir H. Marsh's cases there were collections of a thin pus mixed with curdy matter, one of these in one case communicating with the bowel by an ulcerated perforation, and containing a dark-brown fluid, having a very evident feculent odour. Dr. Gregory observes that the mucous membrane of the bowels, particularly of the small intestines, appears ulcerated in various places, and at these points of ulceration the convolutions of the intestines communicate, so that instead of forming one line of canal, as they will continue to do even in advanced stages of chronic peritonitis, they constitute a mass of tubes communicating freely with each other, and with the thickened and ulcerated peritoneal membrane by innumerable openings.

The prognosis in the majority of cases is unfavorable. Where the peritoneum alone is affected, the patient has certainly a chance of recovery; but if the mesenteric glands, or the mucous membrane of the intestines or pleura be involved, the case will probably terminate unfavorably.

The treatment should consist in the application of leeches to the abdomen, when admissible, poppy fomentations, the use of the warm bath, and the employment of mercury so as to effect the gums. It may be exhibited internally or by inunction; in many cases the latter is preferable, as, when diarrhœa occurs, the bowels are too irritable. A scruple of mercurial ointment should be gently rubbed in over the abdomen, thighs, legs, back, &c., until the gums are touched. Flying blisters to the abdomen are also useful. The diet must be bland and unstimulating.

*Prov. Med. Journ.*

---

### MENINGEAL APOPLEXY.

At the Academy of Medicine, Paris, April 4, 1843, M. Prus read a memoir on this subject, of which the following is the substance:—The author distinguishes this form of apoplexy into two species, viz., sub-arachnoid and intra-arachnoid, and points out the differences between them.

In sub-arachnoid hæmorrhage the blood is frequently derived from a ruptured artery or vein; in twenty-four cases related by the author, this occurred fifteen times, and the rupture of the vessel may have existed in the remaining nine cases. In intra-arachnoid hæmorrhage the effusion of blood always arises from exhalation of that fluid. In the former species the blood is mixed with the cerebro-spinal fluid, and has a constant tendency to pass into the ventricles and vertebral canal; in the latter species the effused blood is generally limited. In sub-arachnoid apoplexy we never find any trace of false membranes; but in intra-arachnoid we always have a false membrane enveloping the clot about the fourth or fifth day.

*Symptoms.*—Muscular paralysis rarely accompanies sub-arachnoid hæmorrhage; it was met with only thrice in twenty-four cases. On the other hand, in eight cases of intra-arachnoid apoplexy, muscular paralysis occurred six times. Loss of sensation, when it does exist, which is very rare, is but slight in both species.

Deviation of the mouth is peculiar to meningeal hæmorrhage. Somnolence and coma almost constantly exist in both. In sub-arachnoid hæmorrhage we have no delirium, fever, or peculiar dryness of the tongue, which belong to the arachnitis which comes on, about the fourth or fifth day, in cases of intra-arachnoid hæmorrhage.

*Termination.*—Sub-arachnoid hæmorrhage was constantly fatal within eight days. In cases of intra-arachnoid apoplexy the patients sometimes lived for thirty days or more; and in some cases a cure is obtained, the blood being absorbed by the inveloping cyst. M. Prus cites an example of this mode of recovery.

*Treatment.*—In sub-arachnoid hæmorrhage the treatment should be directed towards moderating or arresting the effusion of blood, and palliating the effects of pressure. In intra-arachnoid hæmorrhage there are other and important indications to be fulfilled. The formation of the false membrane, which envelopes and may absorb the clot, is not to be interfered with too far, while the effects of arachnitis must be checked. *Ibid.*

---

### ILLEGAL PRACTICE OF MEDICINE IN PARIS.

Some months ago a trial took place before the Tribunal of Correctional Police in Paris, which abundantly shows the jealousy with which the authorities view persons practising medicine in the French metropolis without having previously procured the necessary certificates of competency.

The case was that of a lady in Paris who had been so severely bitten by a dog as to be upwards of a month confined to her bed, and in great suffering, in consequence of which she brought an action against the owner of the dog, and recovered 500 francs damages. The defendent, however, appealed against the verdict on the plea that the original injury had been greatly aggravated by the bad treatment of the medical man whom the lady called in, and proved by a physician's evidence that the remedies used were calculated to irritate instead of allaying the painful symptoms. By these means he succeeded in materially lowering the amount of damages claimed by the lady; and at the same time the Tribunal having decided that the medical man (who was a recognized *officier de santé* for the dep. Saône et Loire) had acted illegally in practising his profession in Paris without special licence for the same, issued an order for his prosecution.—*Lond. Lancet.* April 15, 1843.

